# Genome-Wide Identification and Expression Analysis of WRKY Transcription Factors under Multiple Stresses in *Brassica napus*

**DOI:** 10.1371/journal.pone.0157558

**Published:** 2016-06-20

**Authors:** Yajun He, Shaoshuai Mao, Yulong Gao, Liying Zhu, Daoming Wu, Yixin Cui, Jiana Li, Wei Qian

**Affiliations:** 1 College of Agronomy and Biotechnology, Southwest University, Chongqing 400716, China; 2 Yunnan Academy of Tobacco Agricultural Sciences, Yuxi 653100, China; Huazhong university of Science and Technology, CHINA

## Abstract

WRKY transcription factors play important roles in responses to environmental stress stimuli. Using a genome-wide domain analysis, we identified 287 WRKY genes with 343 WRKY domains in the sequenced genome of *Brassica napus*, 139 in the A sub-genome and 148 in the C sub-genome. These genes were classified into eight groups based on phylogenetic analysis. In the 343 WRKY domains, a total of 26 members showed divergence in the WRKY domain, and 21 belonged to group I. This finding suggested that WRKY genes in group I are more active and variable compared with genes in other groups. Using genome-wide identification and analysis of the WRKY gene family in *Brassica napus*, we observed genome duplication, chromosomal/segmental duplications and tandem duplication. All of these duplications contributed to the expansion of the WRKY gene family. The duplicate segments that were detected indicated that genome duplication events occurred in the two diploid progenitors *B*. *rapa* and *B*. *olearecea* before they combined to form *B*. *napus*. Analysis of the public microarray database and EST database for *B*. *napus* indicated that 74 WRKY genes were induced or preferentially expressed under stress conditions. According to the public QTL data, we identified 77 WRKY genes in 31 QTL regions related to various stress tolerance. We further evaluated the expression of 26 *BnaWRKY* genes under multiple stresses by qRT-PCR. Most of the genes were induced by low temperature, salinity and drought stress, indicating that the WRKYs play important roles in *B*. *napus* stress responses. Further, three *BnaWRKY* genes were strongly responsive to the three multiple stresses simultaneously, which suggests that these 3 *WRKY* may have multi-functional roles in stress tolerance and can potentially be used in breeding new rapeseed cultivars. We also found six tandem repeat pairs exhibiting similar expression profiles under the various stress conditions, and three pairs were mapped in the stress related QTL regions, indicating tandem duplicate WRKYs in the adaptive responses to environmental stimuli during the evolution process. Our results provide a framework for future studies regarding the function of *WRKY* genes in response to stress in *B*. *napus*.

## Introduction

The *WRKY* gene family is one of the most extensively studied transcription-factor gene families in plants [1]. Plant WRKY proteins are characterized by a highly conserved WRKY domain with a 60 amino acid region [2]. It includes the conserved WRKYGQK sequence followed by one of two types of zinc finger motifs, C2H2 or C2–HC [3]. WRKY proteins can be classified into three groups: group I, group II and group III, based on the number of WRKY domains and the types of zinc finger motifs. Group I WRKY contains two WRKY domains and the C2H2-type zinc finger motif (C–X4–5–C–X22–23–H–X1–H). Group II WRKY only contains a single domain and shares the same motif as group I. Group III WRKY contains a single domain and a C2–HC-type motif (C–X7–C–X23–H–X1–C). Group II is further classified into several subgroups based on their phylogenetic clades [3–5].

Since the first plant *WRKY* gene SPF1 was identified in sweet potato [6], numerous *WRKY* family genes have been identified in different plant species. Because whole genome sequences have been completed in numerous plants, *WRKY* family members have been genome-wide identified in several of these species. Previous studies have found 72 *WRKY* family members in Arabidopsis, more than 100 in rice, 57 in Cucumis sativus, 104 in Populus trichocarpa, 81 in Solanum lycopersicum, 197 in soybean, 66 in papaya, 68 in sorghum, 38 in Physcomitrella patens, 35 in Selaginella moellendorii, 80 in Pinus, more than 45 in barley, 56 in Ricinus communis, 119 in maize, 120 in Gossypium raimondii, and 59 in Vitis vinifera [7–12]. Recently, two papers on *WRKY* gene family analysis in the two diploid progenitors of *B*. *napus*, *B*.*rapa* and *B*.*oleracea*, were published, and 145 and 142 *WRKY* genes were detected in the *B*.*rapa* and *B*.*oleracea* genome, respectively [13, 14]. Despite much progress in the identification of *WRKY* genes in plants, to the best of our knowledge, no genome-wide characterization of this gene family has been conducted in *B*. *napus*. The recently released whole-genome sequence in *B*. *napus* and the publicly available *B*. *napus* database will serve as a foundation for identifying the WRKY gene families in *B*.*napus*.

In plants, WRKY transcription factors are known to play prominent roles in plant stress response processes [1, 3]. In the 13 *OsWRKY* genes identified in rice, 11 showed variable responses to salt, polyethylene glycol (PEG), and cold or heat stresses [15]. In the 15 *WRKY* genes detected in wheat, 8 showed responses to cold, heat, NaCl, and PEG treatment [16]. The transgenic *Arabidopsis* plants that over expressed several *GmWRKY* genes of soybean were more tolerant to various stress [17]. To date, 13 WRKY genes have been reported in *B*. *napus* that are associated with stress response processes [18]. Identification of multi-functional roles of *WRKY* genes in stress tolerance may potentially be used to breed new cultivars with increased stress resistance. However, most of the reported *WRKY* genes in *B*. *napus* were only researched under single stress conditions. Co-expression analysis of *WRKY* genes under multiple stresses in *B*. *napus* has not been previously reported.

The objective of this study was to survey the *WRKY* genes in the sequenced genome of *B*. *napus* and to evaluate the expression patterns for several *WRKY* genes under multiple stress conditions. Our work provides a framework for elucidating the structure, evolution and functional roles of *WRKY* genes in *B*. *napus*.

## Materials and Methods

### Identification, classification and motif analysis of the *WRKY* gene family

The genes and proteins annotated in *B*. *napus* were downloaded from http://www.genoscope.cns.fr. WRKY transcription factors were identified using HMMER software version 3.0 [19] and the PFAM protein family database using the WRKY domain (PF03106) as a query [20]. WRKY protein sequences in Arabidopsis were obtained from the Arabidopsis Information Resource (TAIR: http://www.arabidopsis.org/). The MEME program was used to predict the conserved motif [21]. The parameters were set as follows: maximum number of motifs, 10; minimum motif width, six; and maximum motif width, 70. Alignment of the amino acid sequences of the WRKY domain was performed with ClustalX 1.83 [22]. The MEGA 6.0 software was used to construct the phylogenetic tree [23]. A maximum likelihood tree was used based on the bootstrap method. The number of bootstrap replications was 1000.

### Mapping and gene duplication of *WRKY* genes

Positional information about all of the *WRKY* genes was investigated according to the *B*. *napus* information resource database (http://www.genoscope.cns.fr.). The MapChart version 2.2 program was used to map the *WRKY* genes on chromosomes[24]. BIOEDIT software and blast program were used to identify duplicate genes,. A similarity of aligned genes greater than 85% was considered to indicate duplicate genes [25].

### In silico expression analysis of *WRKY* genes

To identify *WRKY* genes with a potential role in response to stress in plants, we analyzed the in silico expression pattern of *WRKY* genes under various stresses. One microarray data set was available in the NCBI database for detecting the patterns of gene expression after inoculating Sclerotinia sclerotiorum. Microarray data were downloaded from the NCBI GEO database (http://www.ncbi.nlm.nih.gov; accession numbers GSM334324–GSM334353, GSM334645–GSM334674). The transcript data were obtained from plant material including five time points: 6, 12, 24, 48, and 72 hours post-inoculation.

For other abiotic stresses, no extensive microarray data for gene expression estimates were found for *Brassica*. Consequently, we used the expressed sequence tag (EST) data from GenBank dbEST to identify *WRKY* genes that were preferentially expressed under each stress condition. All raw ESTs were cleaned by SeqClean (http://compbio.dfci.harvard.edu/tgi/software/) and retained high-quality ESTs for subsequent analysis. EST data were clustered into the different stress conditions according to the tissue source in the EST library description.

### Identification of *WRKY* genes overlapping with known QTLs related to various stresses

The QTL data related to different stresses in *Brassica* were referenced from published papers. According to the physical positions of the flanking markers of the QTLs (http://www.genoscope.cns.fr/blat-server/cgi-bin/colza/webBlat), the corresponding genomic sequences of the QTL region were extracted. Then, the *WRKY* genes residing in these known QTL regions were selected.

### Plant materials and stress conditions

*Brassica napus* accession Zhongshuang11, which exhibits high tolerance to stress, was kindly provided by Oilcrops research Institute, Chinese academy of agricultural sciences, and used for the stress treatments. The Seeds were surface-sterilized in 70% ethanol for 1 min, and then rinsed three times with sterile dH2O. The sterilized seeds were germinated in Petri dishes on two layers of filter papers at 24°C. Three days later, the germinated seedlings were transferred to a MS medium, pH 5.7, containing 0.3% agar and 3% sucrose, and grown under the following conditions: 16/8 h photoperiod, 24°C, 60% relative humidity. Two weeks old plants were exposed to the multiple stresses. The stress conditions included drought, salinity and low temperature. Drought and salinity were applied by immersing the seedlings in 20% PEG-6000 and 200 mmol L^−1^ NaCl, respectively. The cold stress treatment was applied by putting the seedlings under 3°C. The leaves were collected at 0, 3, 6, 9, 12, and 24 hours after the stress treatment, quick-frozen in liquid nitrogen, and stored at −80°C for RNA extraction.

### RNA isolation and real-time PCR analysis

Total RNA was isolated by the RNAprep pure Plant Kit (DP 432) (Tiangen, China) following the manufacturer’s instructions. Each RNA sample was treated with DNase I after the extraction to remove all residual DNA. First-strand cDNA was synthesized using the reverse transcription polymerase reaction system, iScript TM cDNA Synthesis Kit (BIO-RAD, USA). Then, 0.8 μg RNA was reverse transcribed following the instruction manual. The obtained cDNA was diluted to 50 times for qRT-PCR. Primer 5.0 was used to design gene-specific primers for qRT-PCR (http://www.premierbiosoft.com/). The amplified fragment length ranged from 80 bp to 200 bp, and the annealing temperature ranged from 58°C to 65°C. The Arabidopsis Actin7 (AT5G09810) gene was used as the reference gene (forward primer: 5′- TGGGTTTGCTGGTGACGAT -3′, reverse primer: 5′- TGCCTAGGACGACCAACAATACT -3′).

The qRT-PCR was performed using the BIO-RAD real-time PCR system. Amplification was performed under the following conditions, denaturation at 95°C for 10 min, 40 cycles of denaturation at 95°C for 15 s, annealing at 58–65°C for 15 s, and extension at 72°C for 15 s. The default settings were used for the melting curve stage. Three biological replicates, each with three technical replicates, were tested. The gene expression levels were calculated according to Livak and Schmittgen [26].

## Results

### Identification, classification and structural analysis of WRKY family members

To identify the *WRKY* genes in *B*. *napus*, the WRKY domain (PF03106) was used to search the *B*. *napus* genome (See S1 Table). In total, 287 *WRKY* transcription factor genes were identified in the sequenced genome of *B*. *napus* and it represented approximately 0.315% of the whole genome. Among the 287 *WRKYs*, 139 located in the A sub-genome, and 148 located in the C sub-genome. These *WRKYs* represented approximately 0.328% of the A sub-genome and 0.303% of the C sub-genome, respectively. We used the nomenclature system for *BnaWRKYs* to distinguish the *WRKY* genes in *B*. *napus*. Therefore, the *WRKY* genes identified in this study were named from *BnaWRKY001* to *BnaWRKY287*.

**Fig 1:**
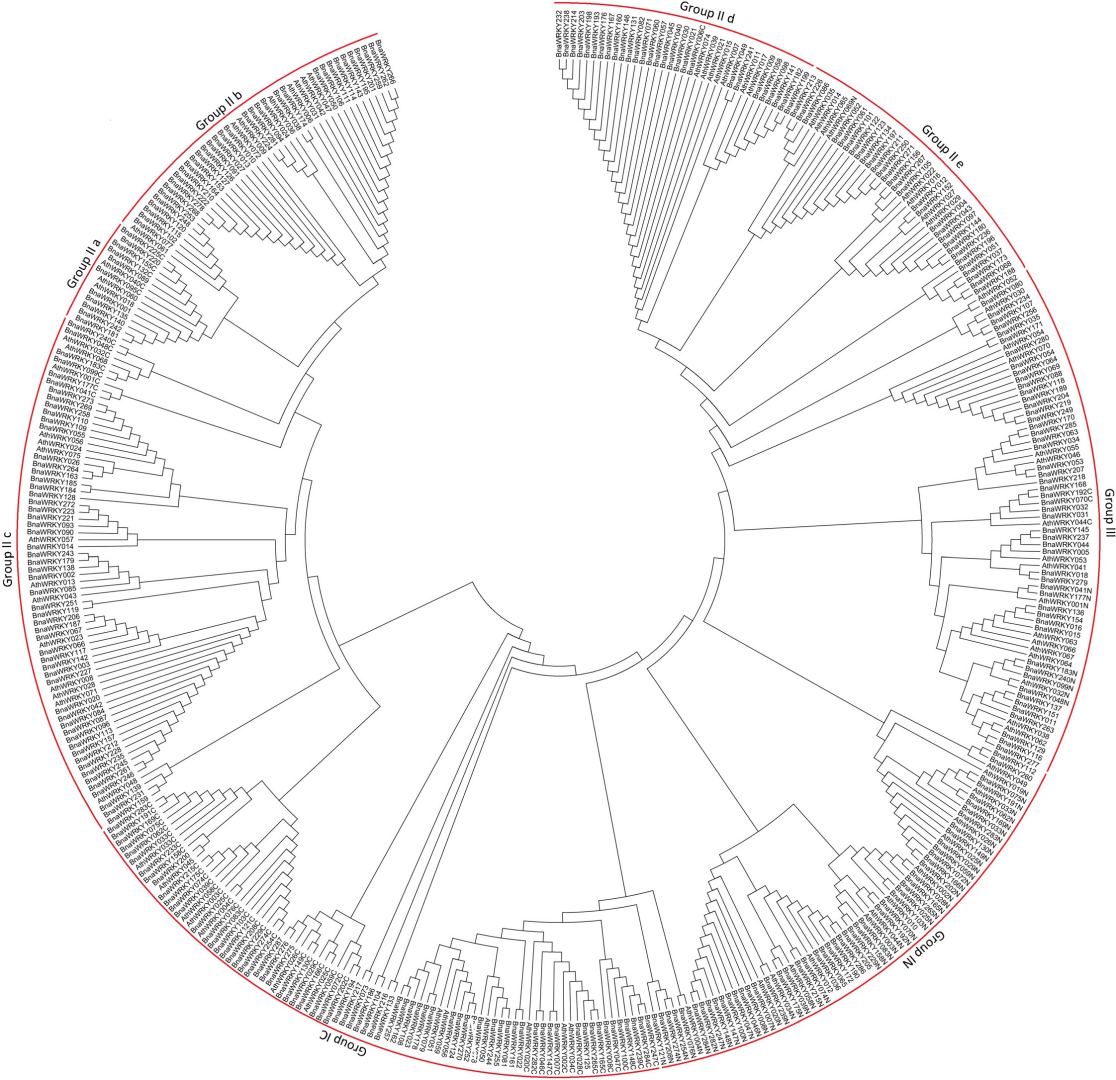
Phylogenetic tree of *Brassica napus* and Arabidopsis *WRKY* genes.

Among the 287 *WRKY* genes, there were a total of 343 WRKY domain regions detected that spanned approximately 60 amino acids (See S2 Table). We found that 56 of the 287 WRKY candidates contained two WRKY domains. The phylogenetic tree was constructed for the *Arabidopsis* WRKYs and *B*. *napus* WRKYs (Fig 1). Based on the classification of the *WRKY* family genes in *Arabidopsis*, the 287 WRKYs with 343 WRKY domains in *B*. *napus* were classified into three groups (groups I, II, and III). Among the three groups, a total of 121 WRKYs belonged to group I, 158 to group II, and 51 to group III. Moreover, the group II genes were further classified into five subgroups (groups IIa–e), containing 11, 34, 55, 28, and 30 WRKY members, respectively. However, the remaining 13 WRKYs were not included in the phylogenetic analysis due to low statistical support. These WRKYs had low identities with other WRKY family members.

**Fig 2:**
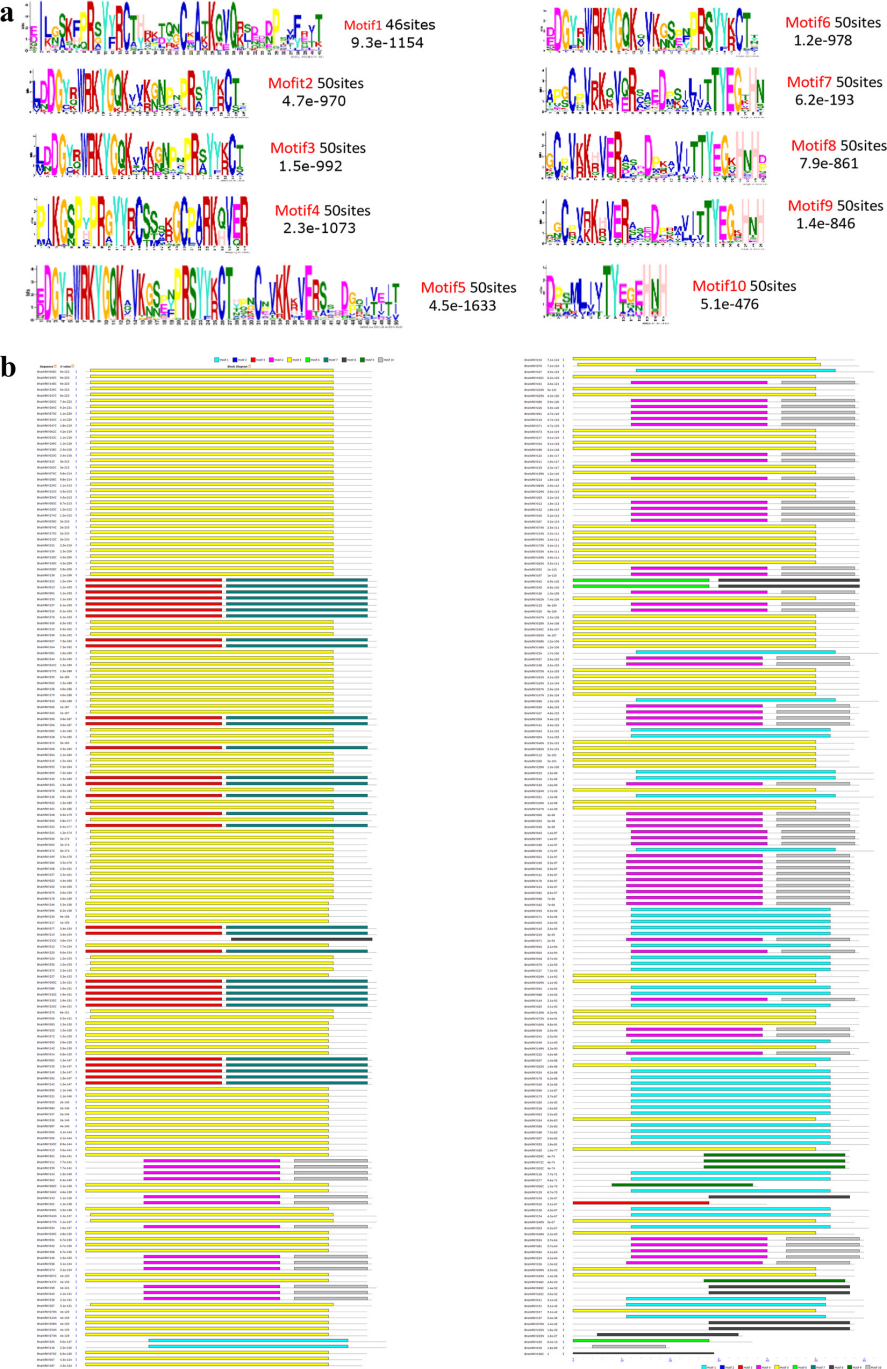
Conserved motifs of *BnaWRKY* members identified using the MEME search tool. a. Logos showing the conserved residues. b. Schematic representation of the related motif at its position in the amino acid sequence. Different motifs are indicated by different colors, and the names of all members and combined p values are shown on the left side of the figure.

**Fig 3:**
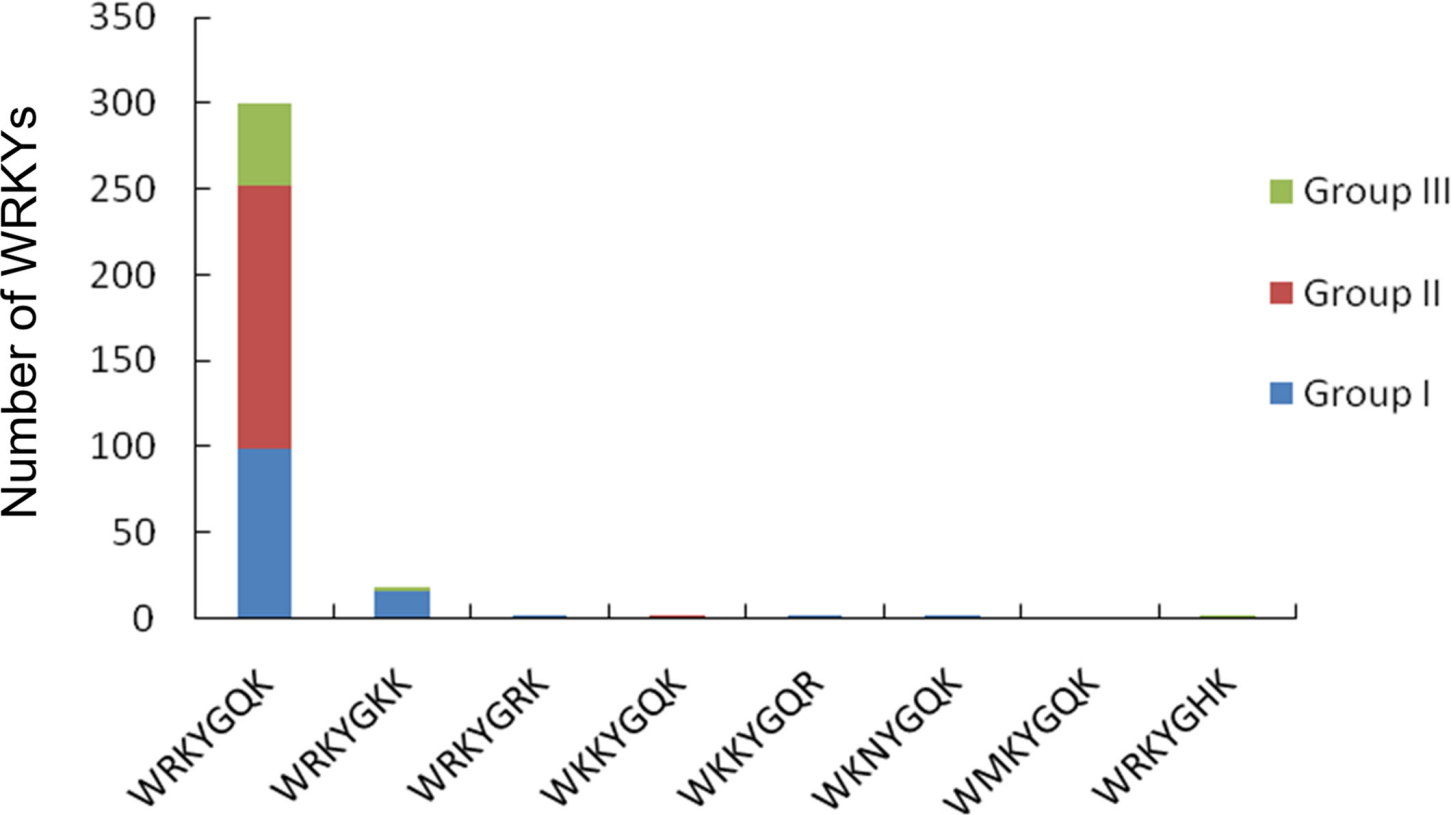
Divergence of WRKY domains in *Brassica napus*. In total, 26 members showed divergence in the WRKY domain in addition to WRKYGQK. Most variations involved Q to K substitutions, 18 of the 26 members belong to WRKYGKK. And in the 26 members, 21 of them belonged to group I.

The 343 WRKY domain regions were subjected to analysis by MEME to reveal conserved motifs shared among related proteins (See S3 Table). Ten conserved motifs, named motifs 1–10, were identified (Fig 2). Among these, the motif encoding the WRKYGQK domain was the most conserved motif identified. In addition to the WRKY domain, the WRKY family members were predicted by MEME to contain other conserved motifs. Alignment of 343 WRKY sequences identified 8 different WRKY motifs. Although the WRKY domain is the most conserved, in addition to WRKYGQK, we found several genes with diverse amino acid residues in this region: WRKYGKK, WRKYGRK, WKKYGQK, WKKYGQR, WKNYGQK, WMKYGQK, and WRKYGHK. In total, 26 members showed divergence in the WRKY domain. Among the seven amino acid residues WRKYGQK, most variations involved Q to K substitutions, 18 of the 26 members belong to WRKYGKK, and within the 26 members, 21 belonged to group I (Fig 3).

### Chromosomal distribution of *WRKY* genes and their genomic duplication

**Fig 4:**
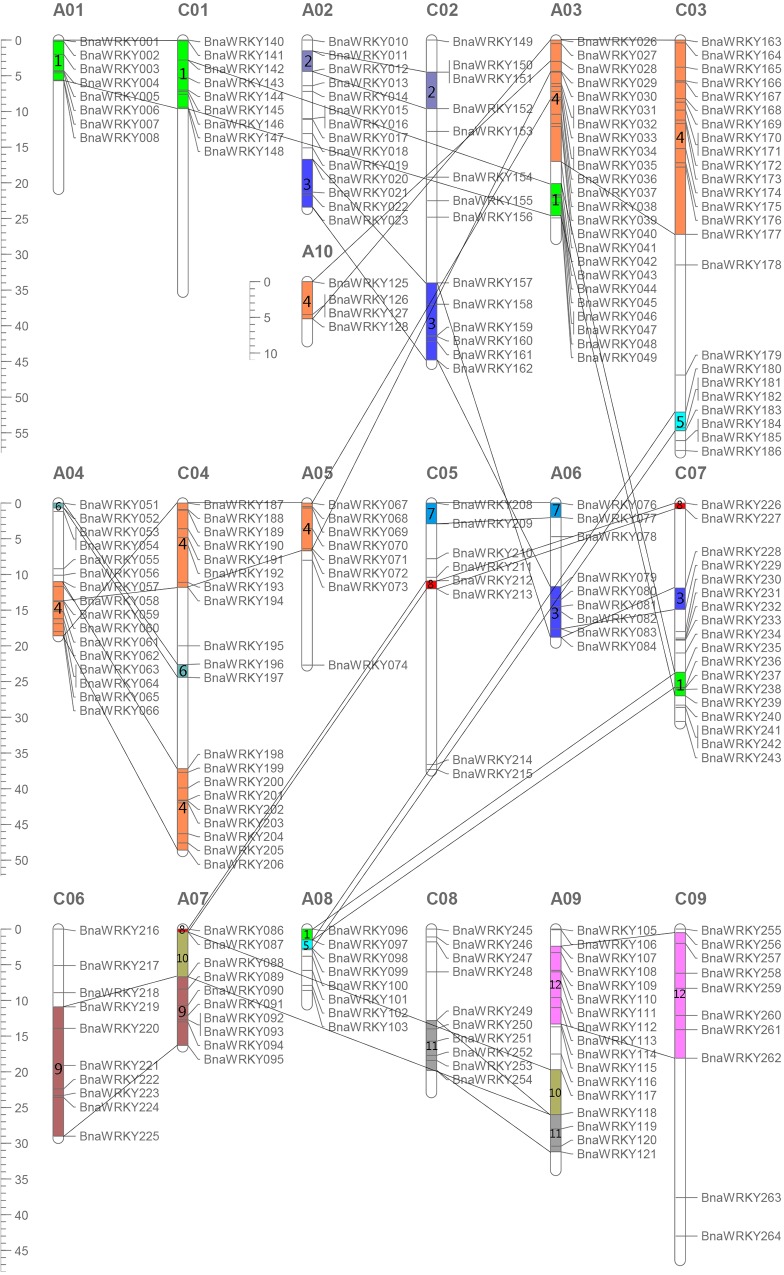
Distribution of *BnaWRKY* genes in *Brassica napus* genome. The chromosomal position of each *BnaWRKY* was mapped according to the *Brassica napus* genome. The chromosome number is indicated at the top of each chromosome. The scale is in mega bases (Mb). The colored bars with numbers on the chromosomes indicate the 12 predicted duplication regions.

**Fig 5:**
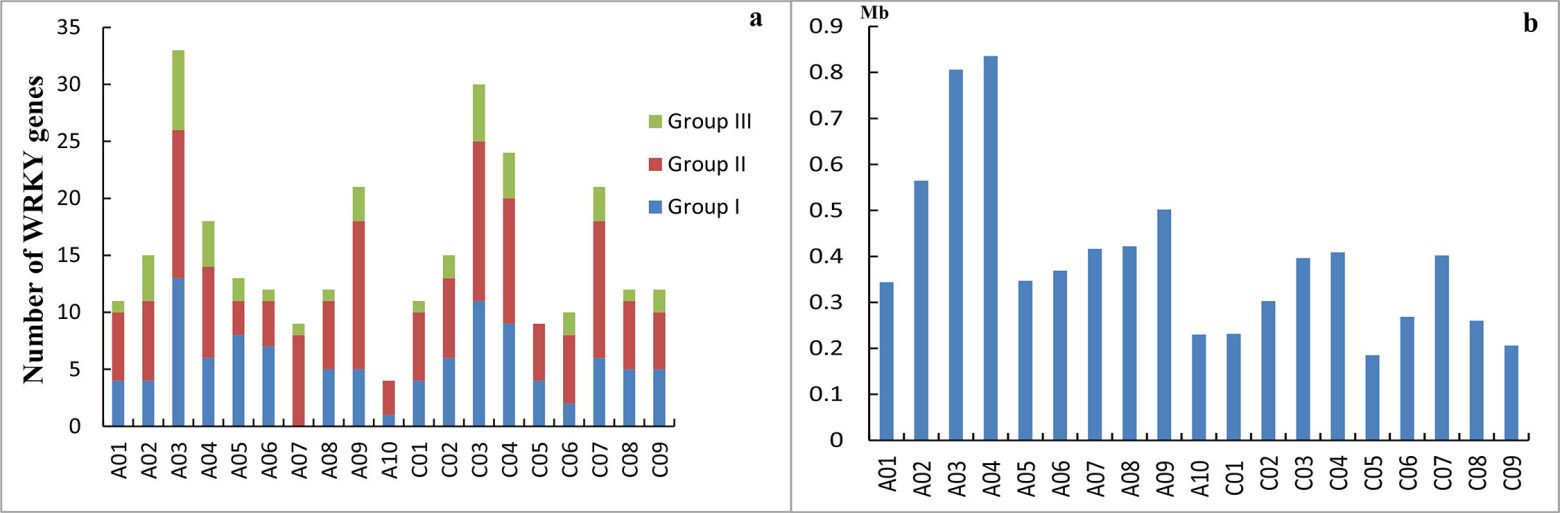
Unevenly Chromosomal distribution of *WRKY* genes in *Brassica napus*. a. Number of *WRKY* genes in each chromosome. b. *WRKY* gene density per chromosome in *Brassica napus*.

To determine the genomic distribution of the *WRKY* genes, the identified *BnaWRKY* genes were mapped on their corresponding chromosome by searching the released database of *B*. *napus*. The results showed that the *BnaWRKY* genes were distributed on all 19 chromosomes (Fig 4); however, the distribution and density of the *WRKY* genes on each chromosome were uneven. There were 8, 14, 24, 16, 8, 9, 10, 8, 17 and 4 *WRKY* genes on chromosomes A1 to A10, respectively, and 9, 14, 24, 20, 8, 10, 18, 10 and 10 *WRKY* genes on chromosomes C1 to C9, respectively (Fig 5A). The other 46 *BnaWRKY* genes mapped onto unanchored scaffolds according to the current database. The *WRKY* gene density per chromosome ranged from 0.185/Mb to 0.835/Mb (Fig 5B). On average, one *WRKY* gene was present every 2.678 Mb. Several chromosomes and chromosomal regions had higher densities of *WRKY* genes compared with others. Chromosome A04 had the highest density of *WRKY* genes, and chromosome C05 had the lowest density.

Distribution of WRKY genes on the chromosomes also showed that within the whole genome of *B*. *napus*, approximately 4.88% (14 of 287) of *WRKYs* were involved in tandem duplication. There were 12 tandem repeats found on the A sub-genome and 2 on the C sub-genome, approximately 8.63% (12 of 139) and 1.35% (2 of 148) of the A sub-genome and the C sub-genome, respectively. In the A sub-genome, these tandem repeats were distributed on Chromosomes A02, A03, A04, A07, and A10 (*BnaWRKY15 and BnaWRKY16; BnaWRKY31 and BnaWRKY32; BnaWRKY34 and BnaWRKY35; BnaWRKY53 and BnaWRKY54; BnaWRKY63 and BnaWRKY64; BnaWRKY126 and BnaWRKY127*). In the C sub-genome, the tandem repeats were distributed on Chromosome C03 (*BnaWRKY170 and BnaWRKY171*) (Fig 4).

*B*. *napus* is a hybrid of *B*. *rapa* (A genome) and *B*. *olearecea* (C genome) [27]. Comparative analysis of *BnaWRKY* gens in the A sub-genome and C sub-genome showed orthologous duplications. Except for 18 *BnaWRKY* genes, we identified 129 and 140 *WRKY* genes in the A and C sub-genomes with orthologous relationships. The orthologous *WRKY* gene pairs tended to be clustered together in the phylogenetic tree. *WRKY* genes in the A sub-genome and C sub-genome were not equally represented within the given clades. For instance, two or more *BnaWRKYs* in the A sub-genome were putative orthologs of a single gene in the C sub-genome. For example, *BnaWRKY015* and *BnaWRKY016* were the orthologs of *BnaWRKY150*.

In addition to gene duplication from the sub-genome-wide polyploidization of the A and C sub-genomes, we observed chromosomal/segmental duplications. Approximately 83% (239 of 287) of the *WRKY* genes were highly similar paralogs. In total, we observed at least 12 potential chromosomal/segmental duplications (Fig 4, pairs of bars with numbers 1–12).

### *WRKY* genes overlapping with known QTLs in *Brassica*

With the QTL data in Brassica, we performed a sequence-based analysis and identified 77 *WRKY* genes within the known QTL regions that were related to various stress tolerances in *Bra*sssica (Table 1) [28–34]. Among these QTL links of *WRKY* genes, several genes were involved in multiple stress tolerances. For instance, *BnaWRKY117* was associated with resistance to *Sclerotinia sclerotiorum* and *Leptosphaeria maculans* (blackleg); *BnaWRKY163* and *BnaWRKY164* were related to diamondback moth and clubroot resistance; *BnaWRKY235* was associated with *fusarium wilt* and clubroot resistance; and *BnaWRKY015* to *BnaWRKY020* were related to *Leptosphaeria maculans* and *Sclerotinia sclerotiorum* resistance. Conversely, we found several *WRKY* genes distributed in the same QTL regions. Interestingly, we also found 3 pairs of tandem duplicate *WRKY* genes in the QTL regions. Tandem repeats of *BnaWRKY15* and *BnaWRKY16* were distributed in the QTL of *Sclerotinia sclerotiorum* and *Leptosphaeria maculans* resistance. *BnaWRKY31*, *BnaWRKY32*, *BnaWRKY34* and *BnaWRKY35* were all distributed in the QTL of *Sclerotinia sclerotiorum* resistance. Our results provide a link between *WRKY* genes and stress resistance in rapeseed breeding and will be useful for genetic improvements in rapeseed.

**Table 1:** *WRKY* genes in stress related QTL regions.

Chr.	QTL name	QTL posiition	*WRKY* genes in QTL region	Stress condition	references
A02	SRA2	900158–10995801	*BnaWRKY010~BnaWRKY014*	Sclerotinia sclerotiorum	Wu et al.2013
A02	SRA2	3808580–20474897	*BnaWRKY012~BnaWRKY020*	Sclerotinia sclerotiorum	Wu et al.2013
A02	qSR10-1	390973–20984604	*BnaWRKY010~BnaWRKY020*	Sclerotinia sclerotiorum	Mei et al.2013
A02	LmA2	9629867–20463672	*BnaWRKY015~ BnaWRKY020*	black leg	Delourme et al.2008
A02	Anju2	4840764–10251601	*BnaWRKY12~BnaWRKY14*	clubroot	Tomita et al.2013
A03	SRA3	5762514–29673240	*BnaWRKY029~BnaWRKY049*	Sclerotinia sclerotiorum	Wu et al.2013
A03	qSR10-2	606874–3131828	*BnaWRKY026~BnaWRKY027*	Sclerotinia sclerotiorum	Mei et al.2013
A06	SRA6	20965425–23324292	*BnaWRKY083*	Sclerotinia sclerotiorum	Wu et al.2013
A08	SRA8	816774–18390028	*BnaWRKY096~BnaWRKY103*	Sclerotinia sclerotiorum	Wu et al.2013
A09	SRA9	2258676–26573318	*BnaWRKY107~BnaWRKY118*	Sclerotinia sclerotiorum	Wu et al.2013
A09	LmA9	17684286–25984575	*BnaWRKY117*	black leg	Delourme et al.2008
C01	qLR09-3	12756747–21935046	*BnaWRKY147~BnaWRKY148*	Sclerotinia sclerotiorum	Mei et al.2013
C01	qLR10-1	12756747–21935046	*BnaWRKY147~BnaWRKY148*	Sclerotinia sclerotiorum	Mei et al.2013
C02	Anju1	42040597–44755227	*BnaWRKY159~BnaWRKY161*	clubroot	Tomita et al.2013
C02	LmC2.1	9837239–16325078	*BnaWRKY152~BnaWRKY153*	black leg	Delourme et al.2008
C02	QTL-1	358639–5209750	*BnaWRKY149*	black rot	Kifuji et al.2013
C03	Anju3	1282758–8855466	*BnaWRKY163~ BnaWRKY167*	clubroot	Tomita et al.2013
C03	QTL-3	411772–5820967	*BnaWRKY163~BnaWRKY164*	diamondback moth	Asghari et al.2009
C04	Sll14a	4910121–9418160	*BnaWRKY191~BnaWRKY192*	Sclerotinia sclerotiorum	Wu et al.2013
C05	LRC5	101013–30986806	*BnaWRKY208~BnaWRKY213*	Sclerotinia sclerotiorum	Wu et al.2013
C05	GC1	6768180–11801042	*BnaWRKY209*	clubroot	Tomita et al.2013
C06	Sll16	7571202–35465622	*BnaWRKY216~ BnaWRKY224*	Sclerotinia sclerotiorum	Wu et al.2013
C06	SRC6.1	31256776–36061993	*BnaWRKY224*	Sclerotinia sclerotiorum	Wu et al.2013
C06	SRC6.2	24665572–35953761	*BnaWRKY221~BnaWRKY224*	Sclerotinia sclerotiorum	Wu et al.2013
C07	qSR10-2	23541376–36743363	*BnaWRKY228~BnaWRKY234*	Sclerotinia sclerotiorum	Mei et al.2013
C07	Anju4	35610814–37812010	*BnaWRKY235*	clubroot	Tomita et al.2013
C07	QTL2(Foc-Bo1)	36671239–39348306	*BnaWRKY235*	Fusarium wilt	Pu et al.2011
C09	qSR09-1	2984476–5282988	*BnaWRKY257*	Sclerotinia sclerotiorum	Mei et al.2013
C09	qSR-09-2	2387812–2861882	*BnaWRKY256*	Sclerotinia sclerotiorum	Mei et al.2013
C09	qLR-09-6	2387812–3087435	*BnaWRKY256*	Sclerotinia sclerotiorum	Mei et al.2013
C09	qSR10-3	2984476–5282988	*BnaWRKY257*	Sclerotinia sclerotiorum	Mei et al.2013

### In silico expression analysis of *WRKY* genes in *B*. *napus* using NCBI databases

High-throughput sequencing and gene expression analyses were performed on *B*. *napus* under both normal and stress conditions. The *B*. *napus* genetic sequences are available in the NCBI database. To identify *WRKY* genes with a potential role in different stress responses in plants, we analyzed the expression pattern of *WRKY* genes in response to various stresses. One microarray data set is available in the *B*. *napus* database and allowed us to compare the differential expression of *WRKY* genes in a partially resistant variety of ZhongYou 821 (ZY821) and a susceptible line of Westar to *sclerotinia*. We therefore examined *B*. *napus* microarray data from different stages after inoculation of *sclerotinia* and collected all of the available *WRKY* gene expression data. In total, we found that 58 *WRKY* genes induced expression by *sclerotinia* (Table 2).

**Table 2:** Preferentially expressed *WRKY* genes under stress tolerance.

EST	*BnaWRKY* Gene	Library/Microarray	Stress condition
BN11150	*BnaWRKY048*, *BnaWRKY240*	GSM334324–GSM334353 GSM334645–GSM334674	Sclerotinia sclerotiorum
BN11784	*BnaWRKY042*, *BnaWRKY245*, *BnaWRKY235*	GSM334324–GSM334353 GSM334645–GSM334674	Sclerotinia sclerotiorum
BN12248	*BnaWRKY028*, *BnaWRKY125*, *BnaWRKY265*,	GSM334324–GSM334353 GSM334645–GSM334674	Sclerotinia sclerotiorum
BN14657	*BnaWRKY059*, *BnaWRKY202*, *BnaWRKY166*	GSM334324–GSM334353 GSM334645–GSM334674	Sclerotinia sclerotiorum
BN14658	*BnaWRKY072*, *BnaWRKY194*	GSM334324–GSM334353 GSM334645–GSM334674	Sclerotinia sclerotiorum
BN14659	*BnaWRKY194*	GSM334324–GSM334353 GSM334645–GSM334674	Sclerotinia sclerotiorum
BN14671	*BnaWRKY113*, *BnaWRKY261*	GSM334324–GSM334353 GSM334645–GSM334674	Sclerotinia sclerotiorum
BN15417	*BnaWRKY005*, *BnaWRKY145*	GSM334324–GSM334353 GSM334645–GSM334674	Sclerotinia sclerotiorum
BN17285	*BnaWRKY169*, *BnaWRKY191*, *BnaWRKY033*	GSM334324–GSM334353 GSM334645–GSM334674	Sclerotinia sclerotiorum
BN18870	*BnaWRKY083*	GSM334324–GSM334353 GSM334645–GSM334674	Sclerotinia sclerotiorum
BN19742	*BnaWRKY009*, *BnaWRKY141*	GSM334324–GSM334353 GSM334645–GSM334674	Sclerotinia sclerotiorum
BN19744	*BnaWRKY049*,*BnaWRKY098*, *BnaWRKY241*	GSM334324–GSM334353 GSM334645–GSM334674	Sclerotinia sclerotiorum
BN19745	*BnaWRKY098*, *BnaWRKY182*	GSM334324–GSM334353 GSM334645–GSM334674	Sclerotinia sclerotiorum
BN20043	*BnaWRKY001*	GSM334324–GSM334353 GSM334645–GSM334674	Sclerotinia sclerotiorum
BN20181	*BnaWRKY055*	GSM334324–GSM334353 GSM334645–GSM334674	Sclerotinia sclerotiorum
BN20309	*BnaWRKY040*	GSM334324–GSM334353 GSM334645–GSM334674	Sclerotinia sclerotiorum
BN22940	*BnaWRKY100*, *BnaWRKY247*	GSM334324–GSM334353 GSM334645–GSM334674	Sclerotinia sclerotiorum
BN23484	*BnaWRKY143*	GSM334324–GSM334353 GSM334645–GSM334674	Sclerotinia sclerotiorum
BN23912	*BnaWRKY095*, *BnaWRKY225*	GSM334324–GSM334353 GSM334645–GSM334674	Sclerotinia sclerotiorum
BN24283	*BnaWRKY061*	GSM334324–GSM334353 GSM334645–GSM334674	Sclerotinia sclerotiorum
BN24410	*BnaWRKY094*, *BnaWRKY224*	GSM334324–GSM334353 GSM334645–GSM334674	Sclerotinia sclerotiorum
BN24459	*BnaWRKY126*,*BnaWRKY127*, *BnaWRKY210*	GSM334324–GSM334353 GSM334645–GSM334674	Sclerotinia sclerotiorum
BN25151	*BnaWRKY007*, *BnaWRKY147*	GSM334324–GSM334353 GSM334645–GSM334674	Sclerotinia sclerotiorum
BN25335	*BnaWRKY064*,*BnaWRKY035*, *BnaWRKY204*,*BnaWRKY189*, *BnaWRKY171*	GSM334324–GSM334353 GSM334645–GSM334674	Sclerotinia sclerotiorum
BN25509	*BnaWRKY118*, *BnaWRKY249*	GSM334324–GSM334353 GSM334645–GSM334674	Sclerotinia sclerotiorum
BN25589	*BnaWRKY082*, *BnaWRKY232*	GSM334324–GSM334353 GSM334645–GSM334674	Sclerotinia sclerotiorum
BN26453	*BnaWRKY050*, *BnaWRKY111*, *BnaWRKY195*,*BnaWRKY259*	GSM334324–GSM334353 GSM334645–GSM334674	Sclerotinia sclerotiorum
BN26664	*BnaWRKY079*, *BnaWRKY178*	GSM334324–GSM334353 GSM334645–GSM334674	Sclerotinia sclerotiorum
BN27460	*BnaWRKY048*	GSM334324–GSM334353 GSM334645–GSM334674	Sclerotinia sclerotiorum
EV194691.1	*BnaWRKY125*, *BnaWRKY265*	dbEST 21489	cold stress
EV218409.1	*BnaWRKY242*	dbEST 21492	drought stress
BG543395.1	*BnaWRKY033*, *BnaWRKY169*	dbEST 8791	Etiolated seedling
BG543470.1	*BnaWRKY033*, *BnaWRKY169*	dbEST 8791	Etiolated seedling
EV113703.1	*BnaWRKY118*, *BnaWRKY249*	dbEST 21479	hydroponically grown root
EV113780.1	*BnaWRKY118*, *BnaWRKY249*	dbEST 21479	hydroponically grown root
EV113862.1	*BnaWRKY118*, *BnaWRKY249*	dbEST 21479	hydroponically grown root
EV113948.1	*BnaWRKY118*, *BnaWRKY249*	dbEST 21479	hydroponically grown root
EV116356.1	*BnaWRKY005*, *BnaWRKY145*	dbEST 21479	hydroponically grown root
EV116444.1	*BnaWRKY005*, *BnaWRKY145*	dbEST 21479	hydroponically grown root
EV117836.1	*BnaWRKY141*, *BnaWRKY009*	dbEST 21479	hydroponically grown root
EV179662.1	*BnaWRKY055*	dbEST 21487	Etiolated seedlings
EV179750.1	*BnaWRKY055*	dbEST 21487	Etiolated seedlings
EV181284.1	*BnaWRKY141*, *BnaWRKY009*	dbEST 21487	Etiolated seedlings
EV181367.1	*BnaWRKY141*, *BnaWRKY009*	dbEST 21487	Etiolated seedlings
EV186271.1	*BnaWRKY199*, *BnaWRKY058*	dbEST 21488	infestation by flea beetles
EV194778.1	*BnaWRKY125*, *BnaWRKY265*	dbEST 21489	cold stress
EV220289.1	*BnaWRKY141*, *BnaWRKY009*	dbEST 21492	drought stress
EV220578.1	*BnaWRKY141*, *BnaWRKY009*	dbEST 21492	drought stress
EV223313.1	*BnaWRKY005*, *BnaWRKY145*	dbEST 21493	insect damage
EV225488.1	*BnaWRKY005*, *BnaWRKY145*	dbEST 21493	insect damage
EX019274.1	*BnaWRKY062*	dbEST 21809	cold stress
EX062868.1	*BnaWRKY191*	dbEST 21814	etiolated mature lea, dark grown
EX063926.1	*BnaWRKY242*	dbEST 21814	etiolated mature lea, dark grown
EX064286.1	*BnaWRKY062*	dbEST 21814	etiolated mature lea, dark grown
EX097528.1	*BnaWRKY033*, *BnaWRKY169*	dbEST 21824	disease
EX120320.1	*BnaWRKY062*	dbEST 21829	defected leaf
EX125680.1	*BnaWRKY191*	dbEST 21831	etiolated mature lea, dark grown

For other stresses, no extensive microarray data for gene expression estimates were found for *B*. *napus*. Consequently, we used the expressed sequence tag (EST) data from GenBank dbEST to identify *WRKY* genes preferentially expressed under stress conditions. In the available ESTs under the various stress conditions, we found that 28 *WRKY* ESTs in 12 different EST libraries were preferentially expressed under different stress conditions, including 3 under drought stress, 3 under cold stress, 7 under a hydroponically grown condition, 10 under a dark condition, 3 infected by insects, and 2 infected by diseases. These ESTs belonged to 16 unigenes (Table 2).

The in silico expression analyses of the *BnaWRKY* genes using public microarray and EST data identified that 74 *BnaWRKY* genes are induced or preferentially expressed under the various stress conditions.

### Expression analysis of WRKY genes under multiple stresses

**Fig 6:**
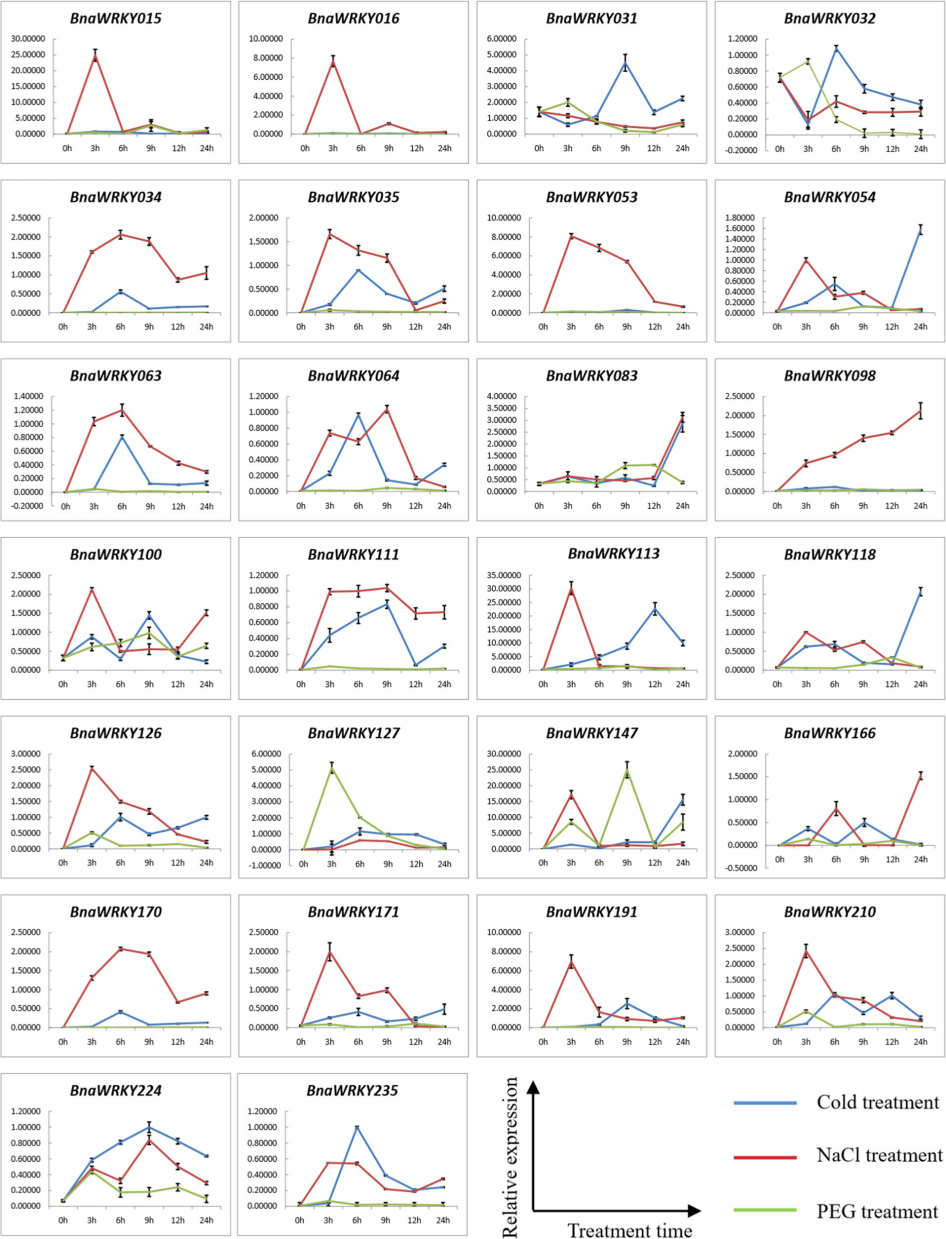
Expression patterns of the 26 *BnaWRKYs* under various abiotic stresses. The Actin7 gene was used as an internal control for qRT-PCR. The y-axis represents relative expression, calculated using the 2^−ΔΔCt^ formula. Expression profiles of *BnaWRKYs* genes under cold (4°C), salinity (200mM NaCl) and drought (20% PEG6000) growth conditions, respectively. Samples were collected at 0, 3, 6, 9, 12 and 24h. *BnaWRKY15* and *BnaWRKY16*, *BnaWRKY31* and *BnaWRKY32*, *BnaWRKY34* and *BnaWRKY35*, *BnaWRKY53* and *BnaWRKY54*, *BnaWRKY63* and *BnaWRKY64*, *BnaWRKY126* and *BnaWRKY127*, and *BnaWRKY170* and *BnaWRKY171* are tandem duplicates, respectively.

Within the 287 *BnaWRKY* genes in the *B*. *napus* genome, 12 were not only identified to be induced or preferentially expressed under stress conditions in silico but also located in the stress related QTL intervals. These 12 *BnaWRKYs* were selected to analyze the expression patterns under multiple stress conditions. Among the 12 *BnaWRKY* genes examined by qRT-PCR, all genes were up-regulated (≥ 2-fold change) under low temperature, salinity and drought stress (Fig 6). The results indicated that the *BnaWRKYs* detected in this study were strongly induced in response to multiple stresses in *B*. *napus*. We also found that *BnaWRKY111*, *BnaWRKY113*, *BnaWRKY118*, *BnaWRKY147*, *BnaWRKY166*, *BnaWRKY191*, *BnaWRKY210* and *BnaWRKY235* were highly up-regulated (≥ 20-fold change) under low temperature stress, *BnaWRKY098*, *BnaWRKY111*, *BnaWRKY113*, *BnaWRKY147*, *BnaWRKY166*, *BnaWRKY191*, *BnaWRKY210* and *BnaWRKY235* were highly up-regulated (≥ 20-fold change) under salinity stress, and *BnaWRKY147*, *BnaWRKY166* and *BnaWRKY210* were highly up-regulated under drought stress (≥ 20-fold change), thus indicating their potential roles in low temperature, salinity, and drought stress, respectively. Additionally, 3 *BnaWRKY* genes, *BnaWRKY147*, *BnaWRKY166* and *BnaWRKY210*, were all highly induced in response to multiple stress treatments (≥ 20-fold change) (Fig 6). Interestingly, the expression processes for several *BnaWRKY* genes exhibited low to high or high to low curve changes over the 24-hour time course. This suggested that the response of *BnaWRKYs* to multiple stresses is a dynamic process.

The 14 tandem duplicate *BnaWRKY* genes in the *B*. *napus* genome were also selected for analysis of their expression profile under multiple stress conditions. Among the 14 *BnaWRKYs* examined by qRT-PCR, most of the genes were up-regulated (≥ 2-fold change), and one pair of tandem repeat genes, *BnaWRKY031* and *BnaWRKY034*, was down-regulated (≥ 2-fold change) under salinity and drought stress (Fig 6). Among the seven pairs of tandem repeats, six pairs including the *BnaWRKY015* and *BnaWRKY016*, *BnaWRKY031* and *BnaWRKY032*, *BnaWRKY034* and *BnaWRKY035*, *BnaWRKY053* and *BnaWRKY054*, *BnaWRKY63* and *BnaWRKY64*, and *BnaWRKY170* and *BnaWRKY171*, exhibited similar expression profiles under the multiple stress conditions, respectively. For example, *BnaWRKY015* and *BnaWRKY016* were significantly induced expressed in response to salinity stress, and their expression trends under the same stress conditions were similar. However, one pair of tandem repeats, *BnaWRKY126* and *BnaWRKY127*, had different expression patterns. *BnaWRKY126* had strongly induced expression with salinity stress, whereas *BnaWRKY127* had strongly induced expression with drought stress (Fig 6).

## Discussion

### Structure, evolution and duplication of WRKY genes in *B*. *napus*

In this study, we used genome-wide data to identify 287 *WRKY* genes, including a total of 343 WRKY domains in *B*. *napus*. Within the 343 WRKY domains, a total of 26 members showed divergence from the WRKY domain, and 21 belonged to group I. This finding suggested that the *WRKY* genes in group I are more active and variable compared with the *WRKY* genes in other groups from *B*. *napus*.

Genome-wide identification and analysis of the *WRKY* gene family in *B*. *napus* identified genome duplication, chromosomal/segmental duplications and tandem duplication. These duplications all contributed to the expansion of the *WRKY* gene family. The number of tandem duplications was much lower than the number of genome and/or segmental duplications, suggesting that whole genome-wide duplication and segmental duplication were major drivers of the *WRKY* gene expansion in *B*. *napus* during the evolutionary process. The 12 segmental duplication bars repeated 2–7 times, suggesting that duplication is not limited to hybridization from the A genome and C genome. This further supported the hypothesis that the whole genome-wide duplication occurred in the two diploid progenitors *B*. *rapa* and *B*. *olearecea* before they combined to form *B*. *napus* [35, 36].

Interestingly, within the 7 pairs of *WRKY* tandem repeats, except for one pair of tandem repeat genes on the C sub-genome, all *WRKY* tandem repeats were on the A sub-genome and belonged to group III, and it was approximately 45% (12/27) of the group III in the A sub-genome. This suggests that *BnaWRKY* genes from group III in the A sub-genome are easy to repeat, and tandem duplication was the main contributor to the enlargement of *BnaWRKY* genes in group III.

### Expression and functional diversity of WRKY genes in *B*. *napus*

The WRKY family is one of the most important transcription factor families and regulates plant responses to biotic and abiotic stresses [1, 3]. In Arabidopsis, rice and soybean, at least 26, 54 and 25 WRKY genes were identified to respond to abiotic stress, respectively [17, 37, 38]. In *B*. *napus*, only 13 *WRKY* genes have previously been reported to participate in defense responses [18]. In this study, we further evaluated the expression of 26 *BnaWRKY* genes under multiple stresses. Most of them were induced by low temperature, salinity and drought stress. These results indicated the WRKYs play important roles in *B*. *napus* stress responses. Notably, 3 *BnaWRKY* genes, *BnaWRKY147*, *BnaWRKY166* and *BnaWRKY210*, were strongly responsive to the three multiple stresses simultaneously. These results indicate that these 3 *WRKY* genes are more likely to be influenced by environmental factors and may have multi-functional roles in stress tolerance. These *WRKYs* may potentially be used for breeding new rapeseed cultivars. Interestingly, the expression processes for several *BnaWRKY* genes exhibited low to high or high to low curve changes over the 24-hour time course. This suggested that the response of *BnaWRKYs* to multiple stresses is a dynamic process.

When using expression analysis for tandem duplication *BnaWRKY* genes, we found six tandem repeat pairs exhibiting similar expression profiles under the various stress conditions, and three pairs were mapped in the stress related QTL regions. These results indicated tandem duplicate *BnaWRKYs* in the adaptive response to environmental stimuli during the evolution process. The duplication of genes may have an important role in maintaining the stability of genetic systems when they are attacked by the external environment [39, 40]. Under natural selection, tandem repeated genes may help organisms adapt to the environment better. A previous study also showed that sorghum to drought tolerance may be related to the duplication of genes [41]. Through expression analysis for tandem duplication *WRKY* genes, we also found one tandem repeat pair showing different expression patterns. The results confirmed that in the evolutionary process of gene expanding, new *BnaWRKY* members may have conservative functions or developed a new and different function. Subfunctionalization and neofunctionalization of the duplicate genes have been confirmed in many species [42–44]. In Arabidopsis, among the tandem repeat pair AtMYB104 and AtMYB81, AtMYB104 is down-regulated by ABA, anoxia and cold stress but is up-regulated under drought, high temperature and salt, whereas the expression pattern of AtMYB81 was the opposite of AtMYB104 [45]. In this study, the differentiations of expression in the tandem repeats indicated their functional diversification.

## Supporting Information

S1 Table Raw output data of *WRKY* genes searched using PF03106 domain. (XLSX)

S2 Table The 287 *WRKY* genes with 343 WRKY domain identified in *B*.*napus*. (XLSX)

S3 Table Raw output data of conserved motifs of *BnaWRKY* members identified using the MEME search tool. (XLSX)
